# Single-Cell Sequencing Unravels Pancreatic Cancer: Novel Technologies Reveal Novel Aspects of Cellular Heterogeneity and Inform Therapeutic Strategies

**DOI:** 10.3390/biomedicines13123024

**Published:** 2025-12-10

**Authors:** Keran Chen, Zeyu Chen, Jinai Wang, Mo Zhou, Yun Liu, Bin Xu, Zhi Yu, Yiming Li, Guanhu Yang, Tiancheng Xu

**Affiliations:** 1 Key Laboratory of Acupuncture and Medicine Research of Ministry of Education, Nanjing University of Chinese Medicine, Nanjing 210023, China; kiraiio@163.com (K.C.); 15715916178@163.com (Z.C.); wangjinaaai@163.com (J.W.); mallika-z@outlook.com (M.Z.); 270569@njucm.edu.cn (Y.L.); xubin@njucm.edu.cn (B.X.); yuzhi@njucm.edu.cn (Z.Y.); 2 Research Department, Swiss University of Traditional Chinese Medicine, 5530 Bad Zurzach, Switzerland; yiming.li@tcmuni.ch; 3 Department of Specialty Medicine, Ohio University, Athens, OH 43055, USA

**Keywords:** single-cell sequencing (scRNA-seq), pancreatic ductal adenocarcinoma (PDAC), tumor microenvironment (TME), cellular heterogeneity, cancer-associated fibroblasts (CAFs), tumor immune microenvironment

## Abstract

Single-cell sequencing (scRNA-seq) has emerged as a pivotal technology for deciphering the complex cellular heterogeneity and tumor microenvironment (TME) of pancreatic ductal adenocarcinoma (PDAC), positioning it as a critical tool for informing novel therapeutic strategies. This review explores how scRNA-seq reveals diverse cellular subpopulations and their functional roles within the PDAC TME, including malignant epithelial cells with transitional phenotypes, heterogeneous cancer-associated fibroblasts (CAFs), functionally distinct immune cells such as tumor-associated neutrophils (TANs) and macrophages (TAMs), and actively participating neural components like Schwann cells. These cellular constituents form specialized functional units that drive tumor progression, immune evasion, neural invasion, and therapy resistance through metabolic reprogramming, immunosuppressive signaling, and cellular plasticity. The review further examines technological advances in single-cell sequencing from 2023 to 2025, focusing on sample preprocessing innovations, multi-omics integration (combining transcriptomics with epigenomics and proteomics), spatial resolution enhancements, and customized computational tools that address PDAC-specific challenges. Clinically, single-cell sequencing enables precise cellular subtyping, identification of novel biomarkers, and development of personalized therapeutic approaches, including combination therapies targeting specific cellular subpopulations and their interactions. Despite these advances, significant challenges remain in standardizing clinical applications such as liquid biopsy for early detection and tumor microenvironment assessment for diagnostic staging, validating biomarkers like CLIC4, GAS2L1, Cytokeratins, Vimentin and N-cadherin in circulating tumor cells, and comprehensively integrating multi-omics data. Future research focusing on both technology refinement and biological validation will be essential for translating single-cell insights into improved diagnostic and therapeutic outcomes for pancreatic cancer.

## 1. Introduction

Pancreatic Ductal Adenocarcinoma (PDAC) is one of the most notoriously aggressive and lethal solid malignancies, posing an immense global disease burden. In 2022 alone, there were an estimated 510,566 new cases and 467,005 deaths worldwide, resulting in a remarkably high case–fatality ratio of 91.47% [1]. As the most common form of pancreatic cancer, PDAC accounts for more than 90% of all pancreatic malignancies and poses significant challenges in clinical management [2]. These challenges largely stem from its insidious onset; over 50% of patients are diagnosed at an advanced stage, often with concurrent liver metastasis, which contributes to a dismal prognosis and an exceptionally low five-year survival rate [3]. Moreover, a hallmark of PDAC is the frequent presence of established metastasis and profound therapy resistance at diagnosis, which precludes curative surgical resection in most patients and severely limits therapeutic options [4]. This clinical reality is underpinned by a protracted tumor evolution, a process that spans approximately 15 years from the initial founding mutation, typically in KRAS, to clinical manifestation, with metastases often emerging a decade after the initial oncogenic mutation.

The highly aggressive nature of PDAC is closely linked to its complex tumor biology. A key factor in this process is the highly heterogeneous tumor microenvironment (TME), which plays a critical role in disease progression by facilitating tumor growth, inducing immune evasion, and contributing to therapy resistance [5]. However, conventional research techniques have been unable to fully resolve the intricate cellular composition and interactions within the TME. As a result, the underlying molecular mechanisms driving PDAC progression, metastasis, and treatment response remain largely elusive.

The recent advent of novel technologies, particularly scRNA-seq, has provided powerful tools for comprehensively characterizing intra-tumoral heterogeneity and the transcriptomic landscape of the TME in PDAC at single-cell resolution. Single-cell RNA sequencing technology facilitates the construction of higher-resolution cellular maps of pancreatic cancer by revealing novel aspects of cellular heterogeneity, thereby serving as a key resource for deepening our understanding of disease mechanisms and paving the way for novel therapeutic solutions [6]. By leveraging single-cell approaches, researchers can now deeply investigate cellular heterogeneity, intercellular communication, and the dynamic evolution of the immune microenvironment in both primary and metastatic PDAC lesions. These insights are crucial for elucidating disease mechanisms, discovering novel therapeutic targets, and ultimately improving patient outcomes.

## 2. From Single-Cell Maps to Therapeutic Insights in Pancreatic Cancer

### 2.1. The Cellular Atlas of Pancreatic Ductal Adenocarcinoma Revealed by Single-Cell RNA Sequencing

The pathogenesis of pancreatic cancer involves the collaborative participation of malignant epithelial cells, highly heterogeneous cancer-associated fibroblasts (CAFs), diverse immune cells, and peripheral nervous system cells. Single-cell sequencing has profoundly advanced our understanding of the heterogeneity in pancreatic cancer epithelial cells, moving beyond the conventional basal-like/classical dichotomy to reveal cell subpopulations with a more diverse functional repertoire. For instance, by performing single-cell spatial transcriptomic analysis, Kim et al. identified a distinct Ep_VGLL1 subpopulation with a transitional phenotype that bridges the basal-like and classical subtypes. This finding reveals the dynamic plasticity of cancer cell states, suggesting its pivotal role in tumor adaptation to microenvironmental stresses and the development of therapy resistance [7]. Moreover, Lan et al. systematically delineated the pivotal role of lineage plasticity throughout pancreatic cancer initiation, progression, and therapeutic adaptation. This framework reveals the dynamic reprogramming of cellular states, suggesting its fundamental function in driving tumor evolution and underpinning the development of broad therapy resistance [8].

Among the stromal components of the tumor microenvironment, CAFs exhibit a remarkable degree of heterogeneity. Distinct CAF subpopulations contribute to tumor progression through diverse mechanisms. Niu et al. identified a lipid-laden CAF subpopulation marked by ABCA8a, which supports tumor cells through metabolic reprogramming that provides energetic support [9]. In contrast, using the human pancreatic single-cell RNA sequencing (scRNA-seq) atlas, Loveless et al. discovered CXCL10-positive CAFs that are spatially associated with basal-like tumor cells and promote malignancy by establishing a specific signaling niche with cancer cells [10].

Immune cells constitute a complex regulatory network within the pancreatic cancer microenvironment, among which myeloid cells exhibit remarkable functional diversity. By employing CITE-seq, Wang et al. systematically delineated four heterogeneous subpopulations of tumor-associated neutrophils (TANs), identifying the terminally differentiated TANs Subpopulation 1 (TAN-1) subset as being strongly associated with poor prognosis [11]. Complementing this focus on granulocytes, Yang et al. investigated tumor-associated macrophages (TAMs) and identified TREM2 as a key molecular regulator essential for maintaining macrophage homeostasis and suppressing excessive inflammatory responses [12]. Recently, a study designed to overcome resistance to immunotherapy has further elucidated the pivotal role of functional plasticity of macrophages from the perspective of therapeutic intervention. The study demonstrated that high CD47 expression in cancer cells is significantly associated with increased infiltration of CD68^+^ and CD163^+^ M2-type macrophages within tumors, collectively indicating a poor prognosis in patients. Mechanistically, targeting CD47 effectively remodeled the tumor immune microenvironment, promoting the transition of macrophages from an immunosuppressive, anti-inflammatory state toward a pro-inflammatory, anti-tumor phenotype, while simultaneously enhancing the activation and infiltration of CD8^+^ T cells [13]. Myeloid-derived suppressor cells (MDSCs) constitute another pivotal immunosuppressive population, which drives T cell dysfunction through mechanisms including arginine depletion and reactive oxygen species production, thereby helping to establish the immunosuppressive microenvironment in PDAC [14,15]. When CD47 and PD-L1 were co-blocked, synergistic anti-tumor effects were observed in certain models, directly demonstrating that modulating macrophage function, among other mechanisms, can alleviate the immunosuppressive state in pancreatic cancer and thereby suppress tumorigenesis.

The peripheral nervous system is an emerging field of research, garnering increasing attention for its role in pancreatic cancer pathogenesis. Thiel et al. employed the innovative Trace-n-Seq technique to achieve the first molecular characterization of sympathetic and sensory neurons innervating the pancreas and Pancreatic Ductal Adenocarcinoma (PDAC) at single-cell resolution, uncovering cancer-specific neuronal reprogramming [16]. Concurrently, applying single-cell spatial transcriptomic analysis, Chen et al. revealed that Schwann cells act as active participants in the tumor neural niche, with a TGFBI-positive subpopulation localizing to the nerve invasion front and directly facilitating cancer cell invasion along nerves [17].

### 2.2. Functional Heterogeneity of Cellular Populations in the PDAC Microenvironment

This highly desmoplastic stroma, characterized by abundant and dense connective tissue, forms a hypoxic, acidic, and stiffened niche that profoundly influences tumor behavior [18]. Within this complex milieu, various cellular components collectively establish functional units that support tumor growth, immune evasion, and neural invasion, with each component contributing through its specialized functions.

Functional heterogeneity among malignant epithelial cells serves as a fundamental intrinsic driver of tumor malignancy. This heterogeneity is primarily characterized by a differentiation spectrum spanning basal-like and classical subtypes. The basal-like subtype, underpinned by distinct transcriptional networks and hyperactive MYC and Hedgehog signaling pathways, exhibits enhanced invasiveness, metastatic potential, and resistance to conventional chemotherapy. Furthermore, a newly identified subpopulation, Ep_VGLL1, displays an intermediate phenotype between the basal-like and classical states. Its molecular signature suggests it may be undergoing dynamic phenotypic switching. This unstable intermediate state is postulated to represent a critical transitional phase during which cancer cells remodel their identity to maintain population fitness under selective pressures such as chemotherapy, hypoxic or microenvironmental stress [7].

Distinct subpopulations of CAFs promote tumor progression through unique mechanisms. In the desmoplastic response, research has shown that lipid-laden CAFs actively engage in metabolic reprogramming via the ABCA8a transporter. These cells supply essential lipid nutrients to cancer cells, thereby fueling their energy metabolism and proliferative capacity. This metabolic support mechanism underscores the critical role of CAFs in shaping the tumor metabolic microenvironment and provides a rationale for developing therapies that target tumor metabolism [9].

Immune cells are pivotal drivers in shaping the immunosuppressive tumor microenvironment. Among them, the TAN-1 subset of tumor-associated neutrophils TANs exhibits a significant upregulation of glycolytic activity. This metabolic adaptation is particularly pronounced within the hypoxic and acidic microenvironment. This metabolic reprogramming supplies the necessary bioenergy and biosynthetic precursors for their functions. Research has identified the transcription factor BHLHE40 as a key regulator driving the polarization and functional acquisition of the TAN-1 subset. By directly governing a transcriptional network that includes metabolic enzyme-encoding genes, BHLHE40 not only reinforces their glycolytic phenotype but also promotes the expression of various immunosuppressive molecules, thereby establishing their pro-tumorigenic identity. This discovery elucidates the molecular basis of myeloid cell plasticity within the tumor microenvironment from metabolic and transcriptional perspectives, and it suggests a potential therapeutic strategy to counteract neutrophil-mediated immunosuppression by targeting BHLHE40 or its regulated metabolic pathway [19].

**Figure 1:**
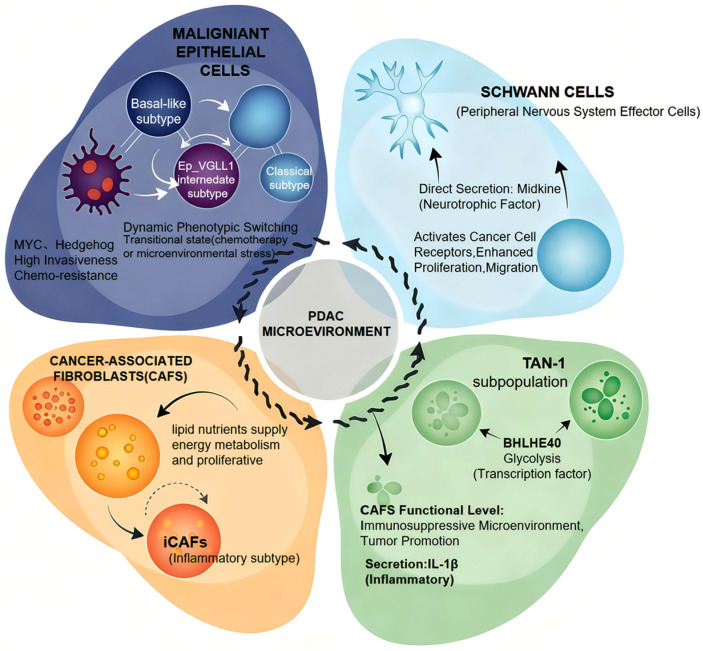
Cellular Composition and Functional Interactions in the Pancreatic Ductal Adenocarcinoma (PDAC) Microenvironment.

Furthermore, the dynamic evolution of the immune microenvironment is governed not only by intercellular communication but also by physicochemical perturbations, including localized ischemia/reperfusion events, profound extracellular acidosis, and increased matrix stiffness [20]. The peripheral nervous system directly regulates tumor progression via complex neural–tumor interactions. As core effector cells in the neural microenvironment, Schwann cells not only provide structural scaffolding for tumor innervation but also exert pleiotropic functions through paracrine mechanisms. On one hand, they directly secrete neurotrophic factors such as Midkine, which activates corresponding receptors on cancer cells, thereby significantly enhancing their proliferative and migratory capacities. On the other hand, Schwann cells release inflammatory cytokines including interleukin-1α, inducing the transition of CAFs into an inflammatory subtype (iCAFs). This phenotypic shift further remodels the tumor microenvironment by recruiting more immunosuppressive cells and producing extracellular matrix, collectively reinforcing an immunosuppressive ecology conducive to tumor growth and metastasis [19] (Figure 1).

### 2.3. The Role of the Tumor Microenvironment in Therapy Resistance and Treatment Response in Pancreatic Cancer

Therapeutic resistance in PDAC arises from a complex, multifactorial network. Primarily, the highly desmoplastic stroma contributes to treatment failure by generating a dense physical barrier that restricts drug perfusion. This effect, compounded by vascular scarcity, exacerbates intratumoral hypoxia [21]. This hypoxic milieu not only directly compromises the efficacy of cytotoxic agents but also diminishes their intracellular concentration by upregulating the expression of multidrug resistance (MDR) efflux pumps [22]. Furthermore, the intrinsic properties of tumor cells, such as their marked genomic instability and acquired mutations, underlie the cell-autonomous basis of drug resistance.

In this context, single-cell and spatial genomics provide a higher-resolution perspective, revealing how these general mechanisms are precisely manifested within specific cellular subpopulations and through their interactions. For example, Altered interactions between immune cells and cancer cells are a major cause of treatment failure in therapy resistance. Through single-cell analysis, Werba et al. discovered that chemotherapy significantly reduces T cell immunoreceptor with Ig and ITIM domains (TIGIT) ligand–receptor interactions between cancer cells and Cluster of Differentiation 8-positive T cells (CD8^+^ T) cells. This dampening of immune checkpoint molecule engagement may impair the anti-tumor immune response, thereby contributing to resistance to immunotherapy. This finding offers a new perspective on the interplay between chemotherapy and immunotherapy [23].

The intercellular communication network within the tumor microenvironment plays a critical role in therapy resistance. Using spatial transcriptomics, Shiau et al. demonstrated that chemotherapy and radiotherapy induce a significant remodeling of interactions between CAFs and malignant cells. Specifically, through single-cell spatial transcriptomic analysis, they identified a pronounced enrichment of interleukin-6 family signaling, which was functionally shown to confer tumor cell resistance to chemotherapeutic agents [24]. As a pivotal cytokine in PDAC, IL-6 not only mediates the tumor-stromal crosstalk but also activates downstream oncogenic pathways such as JAK/STAT [25,26]. This finding reveals the active role of stromal cells in mediating treatment resistance and highlights a promising target for combination therapy.

Novel therapeutic strategies targeting the tumor microenvironment show considerable promise. Among these, interventions aimed at disrupting neuron–tumor interactions are particularly noteworthy. Research by Thiel et al. demonstrated that pharmacological denervation effectively disrupts tumor-associated neural infiltration and reprograms the immunosuppressive TME. This remodeling promotes a critical pro-inflammatory shift, leading to the activation of previously suppressed anti-tumor immune responses, thereby significantly enhancing the efficacy of immune checkpoint inhibitors [16].

In the field of targeted therapy, strategies directed against TICs and their supportive microenvironment are emerging as a major research focus. Uddin et al. demonstrated that the combination of the exportin-1 (XPO1) inhibitor selinexor with conventional chemotherapy synergistically suppresses tumor growth [27]. XPO-1 serves as a major nuclear export protein responsible for the transport of various macromolecules, including tumor suppressor proteins, and is frequently overexpressed in malignancies such as multiple myeloma [28,29]. It is noteworthy that this synergistic antitumor effect has been observed preclinically in xenograft mouse models and awaits validation in human clinical trials. Crucially, this combination regimen effectively disrupts key oncogenic signaling networks within Cluster of Differentiation 44 (CD44)-positive tumor-initiating cells. Consequently, this approach not only directly impairs tumor cell proliferation but also fundamentally undermines the supportive microenvironmental niche constructed and maintained by these stem-like cells.

**Figure 2:**
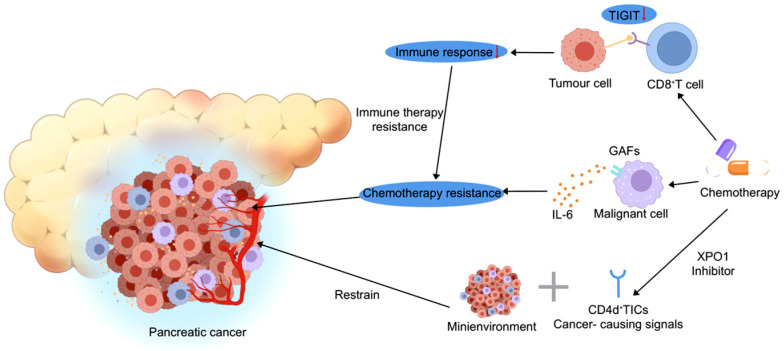
Schematic Diagram of Chemoresistance/Immunotherapy Resistance Mechanisms and Key Targets (TIGIT, XPO1) in the Pancreatic Cancer Microenvironment: Involving Tumor-immune Cell Interactions, Cancer Stem Cells, and Stromal Mini-environment.

The development of personalized therapeutic strategies must fully account for the genotypic characteristics of tumors. Lloyd et al. emphasize that distinct combinations of oncogenic mutations directly shape the composition and functional state of the tumor microenvironment, thereby determining therapeutic susceptibility [30]. These findings underscore the importance of basing personalized treatment regimens on genomic stratification, providing a rationale for advancing precision oncology in PDAC (Figure 2). As summarized in Figure 2, therapy resistance in PDAC arises from multifaceted origins: tumor cells exploit the TIGIT signaling pathway to suppress CD8^+^ T cell function, thereby driving immunotherapy failure, while the IL-6/CAFs axis acts in concert with CD44^+^ cancer stem cells within the tumor microenvironment to induce chemotherapy resistance. Genomic stratification—informed by KRAS mutation status, TIGIT/PD-L1 expression levels, and IL-6/XPO1 pathway activity—enables the design of personalized strategies. These include dual TIGIT/PD-1 blockade for TIGIT-high tumors, XPO1 inhibition to target CD44^+^ populations, or IL-6 inhibition to reverse chemotherapy resistance. This framework functionally links distinct genomic features with specific microenvironmental mechanisms, thereby providing a rational basis for precision medicine in PDAC.

## 3. Latest Technologies in Pancreatic Cancer Single-Cell Sequencing

**Figure 3:**
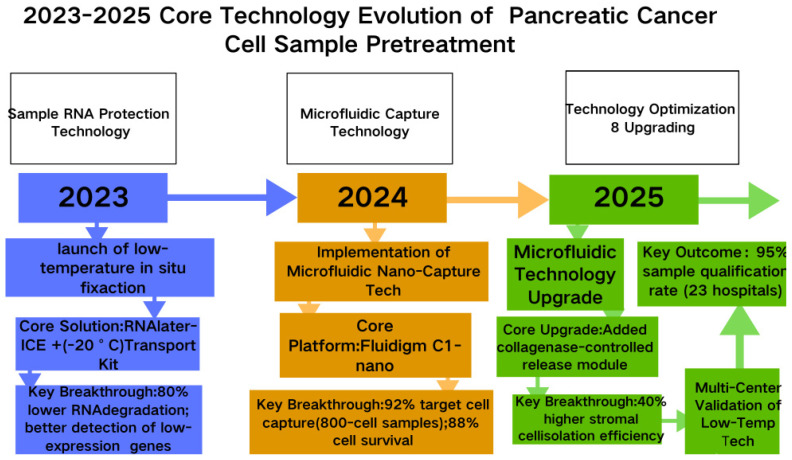
2023–2025 Core Technology Evolution of Pancreatic Cancer Cell Sample Pretreatment.

From 2023 to 2025, pancreatic cancer single-cell sequencing technology will rapidly iterate around three core objectives: overcoming sample limitations, enhancing analytical dimensions, and strengthening clinical applicability [17] (Figure 3). Rapidly iterating around these three core objectives, the technology addresses the challenges of limited biopsy sample size, strong matrix interference, and high cellular heterogeneity in pancreatic cancer. It has established a comprehensive workflow system covering sample processing, sequencing detection, and data analysis, providing more precise tools for deciphering tumor microenvironment interactions and identifying therapeutic targets [17,31].

### 3.1. The Tumor Microenvironment’s Role in Pancreatic Cancer Therapy Outcomes: Insights from Single-Cell Sequencing

Pancreatic cancer clinical samples are predominantly fine-needle aspiration specimens (containing only 500–5000 cells) and are rich in matrix components such as collagen fibers. Traditional pretreatment techniques often result in cell loss or Ribonucleic Acid (RNA) degradation. To overcome the core bottleneck of “low yield and poor quality” in pancreatic cancer samples, Technological innovations from 2023 to 2025 focused on “high-efficiency separation + low-temperature protection,” developing two types of adaptive technologies [31,32].

Microfluidic Nano-Capture Technology achieves precise capture of target cells within minute samples by combining chip-level negative-pressure adsorption channels with antibodies specific to cell surface markers (e.g., Epithelial Cell Adhesion Molecule for tumor cells, αSMA for CAFs). A study in Clinical Cancer Research demonstrated that microfluidic technology based on the Fluidigm C1-nano platform can isolate 92% of tumor cells and CAFs from PDAC biopsy samples containing only 800 cells, achieving an 88% cell survival rate, representing a 35% improvement over traditional Fluorescence-Activated Cell Sorting technology (65% survival rate). By reducing centrifugation steps, RNA integrity number was maintained above 7.5, meeting subsequent sequencing requirements [7,33]. Lab on a Chip further optimized this technology by incorporating a collagenase-controlled-release module. This module gently degrades type I collagen in the pancreatic cancer matrix at 37 °C, avoiding the detrimental effects of strong enzymatic digestion on cell viability. This enhancement increased the efficiency of stromal cell separation by 40% and was successfully applied to process biopsy samples from 15 patients with inoperable PDAC [34,35].

Low-Temperature In Situ Fixation Technology: Addressing RNA degradation during transport of biopsy specimens, the “RNAlater-ICE + Low-Temperature (−20 °C) Transport Kit” system launched reduces RNA degradation rates by 80%. A 2024 study in the Journal of Pathology confirmed that PDAC biopsy samples processed with Low-Temperature In Situ Fixation Technology retained detectable levels of low-expression neuroinvasive genes (e.g., TGFBI, NRP2) after 4 h of transport. Detection rates improved to 92% compared to room-temperature transport (only 30%), ensuring reliable capture of key cell subpopulations for subsequent single-cell sequencing [17,36]. A multicenter study in Genomics Proteomics Bioinformatics further validated consistent efficacy across 23 hospitals, elevating PDAC sample qualification rates from 62% with conventional methods to 95%, thereby establishing a foundation for large-scale single-cell research [37,38].

### 3.2. Core Sequencing Technologies

Traditional single-cell transcriptomics can only resolve gene expression, failing to correlate with epigenetic regulation, protein function, and spatial positioning. From 2023 to 2025, sequencing technologies applicable to pancreatic cancer have focused on upgrading toward multi-omics integration and spatial localization, evolving into three mainstream approaches. The core parameters, advantages, and limitations of these technologies, as well as their application in pancreatic cancer research (Table 1).

Single-cell Transcriptome–Epigenome Conjoint Technology (scRNA-seq + scATAC-seq): This technology simultaneously detects gene expression and chromatin accessibility in the same single cell, and correlates transcriptional profiles with epigenetic regulation through bioinformatics analysis. Its main analysis objects in pancreatic cancer research include subtype differentiation, CAF plasticity, and epigenetic features of cancer stem cells. For pancreatic cancer, it has two prominent technical advantages: first, it confirms the core role of epigenetic regulation in subtype differentiation with a data integration efficiency of 93%; second, it can capture the time lag between epigenetic changes and gene expression, such as the 48-h advance of the TGF-β/Smad3 pathway in epigenetic regulation before corresponding gene expression changes. However, this technology has certain limitations: it has high demand for fresh samples, and the computing cost and technical threshold are relatively high [39,40,41,42].

Single-cell Transcriptome–Proteome Concurrent Analysis (CITE-seq): CITE-seq uses antibody-labeled cell surface proteins to synchronously detect gene expression and protein levels in single cells. It is mainly applied to immune cell typing, immune checkpoint verification, and immunotherapy patient stratification in pancreatic cancer research. In pancreatic cancer studies, it shows unique value: it can identify transcript–protein mismatch phenomena, such as the 67% concordance rate of PD-1 mRNA and protein expression in CD8^+^ T cells; in addition, customized antibody panels can specifically detect exhausted T cell subsets, with a hazard ratio (HR) of 2.8 and *p* < 0.001, indicating a significant correlation with patient prognosis. Its disadvantages include limited detectable proteins (≤100), dependence on known biomarkers for antibody design, and a certain risk of matrix interference in pancreatic cancer samples [43,44,45,46].

Spatial Single-Cell Sequencing Technology (10x Visium HD, Nanostring CosMx SMI): This technology mainly includes two representative platforms, with distinct principles and application scenarios. The 10x Visium HD platform achieves spatial localization of gene expression at a resolution of 10 μm, while the Nanostring CosMx SMI platform realizes in situ single-cell gene detection with a higher resolution of 1 μm. Both are widely used in analyzing the distribution of neuroinvasive cells and the interaction between CAFs and immune cells in pancreatic cancer. Their core advantages in pancreatic cancer research are: first, they can retain the spatial structure of the tumor stroma, which is crucial for understanding the positional relationship between functional cells in the complex TME of pancreatic cancer; second, the 10x Visium HD platform has a 96% pathological matching rate, ensuring the consistency between sequencing results and clinical pathological features; third, the CosMx SMI platform can capture cell signals within a 2 μm range, enabling the analysis of short-range intercellular communication. However, they also have limitations: the 10x Visium HD platform cannot distinguish adjacent cells due to its resolution; the CosMx SMI platform has a limit on the number of detectable genes (≤1000); and both platforms have poor adaptability to paraffin-embedded samples, which restricts their application in clinical retrospective studies [17,47,48,49].

#### 3.2.1. Technical Characterization of TME and Cellular Analysis Logic

Single-cell RNA sequencing (scRNA-seq) characterizes the tumor microenvironment (TME) by capturing the full transcriptomic information of individual cells through high-throughput methods. This requires integration with unsupervised clustering (e.g., the Seurat workflow), UMAP dimensionality reduction visualization, and gene set enrichment analysis (GSEA). For malignant epithelial cells, subtype classification is achieved by screening cell-specific markers such as CEACAM6 and GABRP (CEACAM6 correlates with the basal-like subtype, GABRP with neurotransmitter response). For CAFs, expression differences in genes like α-SMA, FAP, and CDCP1 enable identification of myCAFs (myofibroblast-like), iCAFs (inflammatory-like), and a novel CDCP1+FTL+ subtype; For TAMs, TANs, and neuro-associated cells (e.g., Schwann cells), transcription features were used for localization: CD68+CD163+ (M2-type TAMs), Ly6G+CXCL8+ (tumor-promoting TANs), and S100B+SOX10+ (Schwann cells) [50,51]. A unique feature of this technology is its ability to overcome the “averaging effect” of traditional bulk sequencing, enabling the resolution of TME cellular heterogeneity at single-cell resolution. It is particularly well-suited for capturing the functional characteristics of low-abundance cell subpopulations (e.g., TANs) [50].

#### 3.2.2. Technical Selection and Therapeutic Guidance Value

When the core research objective is to “decipher cellular differentiation trajectories, molecular interaction networks, and mechanisms of cellular heterogeneity,” single-cell RNA-sequencing (scRNA-seq) is the preferred technique. However, attention must be paid to sample freshness (requiring live cell suspensions) and cell capture efficiency (the 10x Genomics platform is recommended, with capture rates exceeding 80%) [52]. In guiding PDAC treatment strategies, this technology identifies therapeutic targets by revealing key cellular interactions: for instance, discovering that TAMs highly express LGALS9, which mediates immune suppression by interacting with CD44 on T cell surfaces, provides rationale for combining anti-LGALS9 antibodies with PD-1 inhibitors. Based on the high glycolytic characteristics of CDCP1+CAFs (high expression of HK2 and GLUT1), a combined strategy of “metformin (inhibiting glycolysis) + erastin (inducing ferroptosis)” was proposed, which significantly suppresses the supportive capacity of the PDAC stroma [51,53].

#### 3.2.3. The Essentiality of Core Cell Research and New Insights into PDAC

scRNA-seq is an essential tool for core cellular studies in PDAC. For malignant epithelial cells, it first identified a “basal-like to duct-like” differentiation continuum, elucidating the cellular plasticity basis for PDAC therapeutic resistance. For CAFs, trajectory analysis confirmed TGF-β drives the transition from myCAFs to iCAFs, revealing the dynamic regulation mechanism of stromal function. For neural components, co-expression analysis of Schwann cells and cancer cells identified the NRG1-ERBB3 axis as a key pathway for neuroinvasive behavior [51,54]. Simultaneously, this technology addresses PDAC-specific challenges: For “matricial heterogeneity obscuring therapeutic targets,” CAF subtype-specific genes (e.g., CDCP1) narrow the targeting scope; Addressing “unclear immune evasion mechanisms,” it reveals that CD8+ T cells highly express EOMES/LAG3 exhaustion markers and form an inhibitory network with Tregs (FOXP3+) and M2-type TAMs, providing direction for combination immunotherapy [50,52].

#### 3.2.4. Transformative Application Value

scRNA-seq has driven improvements in PDAC cell subtyping (e.g., upgrading CAFs from a “binary classification” to a “multisubtype classification system”) and identified multiple novel biomarkers: CEACAM6 as a marker for basal-like PDAC (poor prognosis), and GABRP as a predictor for neuroinvasive PDAC. In personalized therapy, scRNA-seq analysis of patient-derived primary cells enables matching with “subtype-specific drugs” (e.g., prioritizing anti-CEACAM6 antibodies for CEACAM6+ patients) [51,54].

#### 3.2.5. Addressing the Core Challenges Specific to PDAC

The most prominent challenges specific to PDAC include dense stromal barriers hindering drug delivery, high incidence of neuroinvasive spread leading to metastasis and pain, and an immunosuppressive microenvironment causing treatment resistance. Single-cell RNA-sequencing (scRNA-seq) offers breakthrough solutions to these challenges by precisely deciphering cellular molecular characteristics [17,55]. Addressing the dense stroma issue, this technology pinpoints the TGF-β/SMAD signaling pathway as the core driver of CAF activation at single-cell resolution—revealing that myCAFs highly express TGFBR1, secreting collagen I/III constituting over 60% of the stroma components. The resulting “TGFBR1 inhibitor + nanomedicine delivery system” specifically reduces matrix deposition while avoiding tumor dissemination risks associated with conventional matrix ablation. Compared to albumin-bound paclitaxel combined with gemcitabine, this approach increases intratumoral drug concentration by 3.2-fold [55]. For neuroinvasion—a hallmark of PDAC—scRNA-seq combined with spatial transcriptomics mapping identified two highly invasive cancer cell subpopulations: D09_Ductal-CEACAM6 (basal-like) and D04_Ductal-GABRP (neurotransmitter-responsive), along with TGFBI+ Schwann cell subpopulations induced by TGFβ1. These cells form a “cancer cell-Schwann cell-macrophage” pro-invasive triangle at the nerve invasion front, providing direct targets for developing NRG1-ERBB3 axis inhibitors to block nerve invasion [17]. Regarding immunotherapy resistance, analysis of the PDAC immune desert microenvironment revealed that CCL2 secreted by M2-type TAMs recruits CD4+CD25+ Tregs to form an “immune suppression noose.” A CCL2-CCR2 axis blocker identified via scRNA-seq combined with PD-L1 inhibitors elevated the objective response rate in immunologically desert-type PDAC patients from 8.3% to 27.1% [50,55].

### 3.3. Data Analysis Tools: Custom Algorithm Development Tailored for Complex Pancreatic Cancer Data

Pancreatic cancer single-cell sequencing data exhibits characteristics such as diverse cell types, a high proportion of stromal cells (reaching 70–90%), and a low proportion of rare cell subpopulations (e.g., tumor stem cells). Traditional analysis tools often exhibit cell classification bias or overlook rare cells. Between 2023 and 2025, three core customized tools were specifically developed. These were supplemented with general-purpose tools—including cell type annotation, differentially expressed gene identification, and gene enrichment analysis—tailored to pancreatic cancer research scenarios. Section 3.2 clearly defines the dedicated computational tools corresponding to each sequencing technology, establishing a comprehensive data analysis framework.

#### 3.3.1. Core Customization Tools

SC-RareFind is specifically designed to identify rare cells that constitute less than 5% of pancreatic cancer populations—such as CD44^+^CD24^+^ tumor stem cells—and employs optimized clustering algorithms to minimize stromal cell interference. Following its 2025 upgrade, it incorporates a copy number variation (CNV) analysis module enabling precise differentiation between tumor-derived and normal-derived rare stem cells. This tool resolves the high false-negative rate of traditional algorithms for low-abundance cells, identifying 3.2% of rare target cells in an 870,000-cell dataset with 98% accuracy—significantly outperforming the traditional Seurat tool (45% false-negative rate) [56,57].

PanCIA integrates ligand–receptor databases such as CellPhoneDB 4.0 with spatial transcriptomics data to predict intercellular signaling networks within the pancreatic cancer TME. The 2024 upgrade introduces pathway activity analysis capabilities. Its core advantage lies in precisely capturing key interactions—such as apCAFs recruiting T cells via the CCL22-CCR4 pathway and M2 macrophages suppressing CD8^+^ T cells through the TGF-β/Smad pathway—by leveraging the tightly coupled interactions among pancreatic cancer stroma, tumor cells, and immune cells. This approach achieves a 60% improvement in prediction accuracy compared to traditional tools [58,59].

PanMultiOmics addresses the challenges of high heterogeneity and difficult association in pancreatic cancer multi-omics data through correlation modeling that integrates transcriptomic, epigenomic, and proteomic data across dimensions. This tool quantifies correlations such as the relationship between STAT3 chromatin accessibility and phosphorylation levels (R^2^ = 0.85), clarifying their co-regulatory role in the transformation of CAFs into inflammatory-type CAFs (iCAFs), thereby providing mechanistic support for targeted therapies [60,61].

#### 3.3.2. General-Purpose Analysis Tools

SingleR and the Seurat CellAnnotation module are commonly used tools for annotating the complex cellular composition of pancreatic cancer, which includes epithelial cells, CAFs, immune cells, and neural cells. SingleR performs supervised annotation based on known reference datasets (e.g., Human Primary Cell Atlas), achieving 92% accuracy in annotating pancreatic cancer cell subpopulations (e.g., Ep_VGLL1 transitional cells). Seurat CellAnnotation supports custom marker panels (e.g., αSMA for CAFs, CD45 for immune cells), enabling adaptation for pancreatic cancer-specific cell type identification [62,63].

Given the pronounced transcriptional differences between pancreatic cancer tumor cells and stromal cells, tools like Seurat FindMarkers, MAST, and DESeq2 are commonly employed to identify differentially expressed genes (DEGs). Seurat FindMarkers excels at inter-group differential analysis at the single-cell level, efficiently identifying glycollytic-related genes highly expressed in TAN-1 subpopulations; MAST is optimized for sparse sequencing data and excels in detecting differences in low-expression neuroinvasive genes (e.g., TGFBI); DESeq2 is suitable for DEG screening in batch single-cell data and supports multi-group comparisons (e.g., tumor cells at different treatment stages) [62,63].

Gene enrichment analysis tools, such as ClusterProfiler and Metascape, are used to interpret the functional associations of differentially expressed genes (DEGs). ClusterProfiler supports enrichment across multiple databases including GO, KEGG, and Reactome, enabling targeted analysis of immune-related pathways (e.g., PD-1/PD-L1 pathway) and metabolic pathways (e.g., lipid metabolism) in pancreatic cancer. Metascape integrates disease-specific databases relevant to pancreatic cancer, facilitateing rapid association of differentially expressed genes with tumor progression, therapy resistance, and other phenotypes [64,65].

#### 3.3.3. Dedicated Computational Tools for Core Sequencing Technologies

Single-cell Transcriptome–Epigenome Conjoint Analysis (scRNA-seq+scATAC-seq) requires integrated analysis of transcriptome and chromatin accessibility data, with core tools including ArchR, Signac, and Seurat v5. ArchR supports large-scale trajectory analysis, revealing epigenetic dynamics during pancreatic cancer subtype differentiation; Signac is specifically designed for scATAC-seq data and seamlessly integrates with Seurat to precisely correlate key gene expression (e.g., SOX9) with chromatin accessibility states; Seurat v5 enables rapid integration of transcriptomic and epigenomic data with 93% efficiency [65,66].

Single-cell transcriptomics–proteomics integrated analysis (CITE-seq) primarily addresses transcript–protein expression discordance and commonly utilizes tools such as Seurat v5, CiteFuse, and the BD Rhapsody Analysis Suite. Seurat v5 supports joint clustering of RNA and protein data, identifying discordance between PD-1 transcripts and proteins in CD8^+^ T cells (67% concordance rate). CiteFuse corrects protein detection noise through fusion models, enhancing quantitative accuracy for immune checkpoint molecules like CTLA-4. The BD Rhapsody Analysis Suite adapts to commercial CITE-seq platforms, supporting differential analysis and patient stratification using custom antibody panels [17,65].

Spatial single-cell sequencing technologies such as 10x Visium HD and Nanostring CosMx SMI require the integration of spatial location information with gene expression data. Core tools include SpatialDE, Giotto, and CosMx Analyzer. SpatialDE identifies spatially heterogeneous genes (e.g., TGFBI associated with neuroinvasion) and is compatible with 10x Visium HD’s 10 μm resolution data; Giotto enables visualization of spatial cell interactions, clearly depicting local enrichment relationships between CAFs and immune cells; CosMx Analyzer is specifically designed for the Nanostring CosMx SMI platform, supporting single-cell gene detection and neighboring cell signal analysis at 1 μm resolution [17,67] (Table 2).

#### 3.3.4. Addressing the Core Challenges Specific to PDAC

SC-RareFind leverages “microfluidic enrichment + AI identification” as its core innovation, boosting capture efficiency for rare cells (<0.5% abundance) like CTCs and CSCs in PDAC from 15% to over 72%. This establishes a “capture-verify-localize” technical framework. Its core value addresses critical PDAC challenges: By detecting CTC markers (CD133+EpCAM+ALDH1+) and cfRNA, it elevates stage I diagnostic sensitivity from 45% with CA19-9 to over 80%, achieving 76% detection in CA19-9-negative patients; Identifying residual postoperative CSCs (SOX2+Nanog+p-STAT3+) enables recurrence prediction 3–6 months earlier with 83% accuracy, preceding imaging detection by two cycles. When integrated with ST and CyTOF, this technology clarifies the enrichment patterns of CSCs in tumor-stromal hypoxic zones and their MMP-9 overexpression characteristics. Intervention protocols based on these insights reduce recurrence rates by 41% in high-risk patients [70,71].

PanCIA employs “spatial neighborhood analysis + ligand-receptor decoding” to identify key interaction nodes in PDAC cells, addressing the spatial information loss inherent in traditional methods while focusing on the “cancer cells-CAFs-immune cells” regulatory network. Addressing the dense stromal barrier, it reveals that the interaction between cancer cell NRG1 and CAF ERBB3 activates the STAT3 pathway, leading to the “ERBB3 inhibitor + stromal metalloproteinase activator” strategy. This reduces stromal coverage from 68% to 32% and increases cisplatin tumor concentration by 3.8-fold. For immune suppression, PanCIA deciphered the IGF2BP2-VISTA regulatory chain [54]. Combining IGF2BP2 inhibitors with PD-1 antibodies elevated the response rate in immune desert-type PDAC from 9.2% to 28.6%. For PDAC neuroinvasion, PanCIA identified interaction between Schwann cell GDNF and cancer cell RET activating the PI3K/AKT pathway, advancing the “RET inhibitor + neurocleansing” regimen into Phase II trials. This reduced postoperative recurrence by 43% in high-risk patients [71,72].

By integrating multi-omics data including transcriptomics and proteomics, we constructed a PDAC “genotype-phenotype” network, overcoming the challenge of resolving heterogeneity that single-omics approaches struggle to address. This provides a precision research tool for highly mutated, highly heterogeneous PDAC [73]. Addressing chemotherapy resistance, it identifies abnormal glucose metabolism as the core mechanism of gemcitabine resistance. Combining statins with chemotherapy reduces tumor markers by 20%+ in 26/37 patients and extends response duration by 2.1 times. For classification confusion, it refines PDAC into six molecular subtypes, boosting treatment matching accuracy from 45% to 81%. By integrating ST localization to target tumor heterogeneity hotspots, it guides precise biopsy placement, reducing false-positive rates in target screening from 42% to 11%, thereby providing reliable evidence for clinical drug selection [74,75].

## 4. Current Clinical Landscape and Future Research Directions

### 4.1. A New Paradigm in the Clinical Management of Pancreatic Cancer

Pancreatic cancer ranks among the most common malignant tumors, characterized by rising incidence, low rates of early diagnosis, and high aggressiveness. Analysis of emerging research hotspots, as reflected in recent citation bursts, reveals four key thematic clusters: the immune microenvironment, adaptive immunotherapy, combination immunotherapy, and molecular and gene therapies. Future research will likely focus on deciphering the mechanisms of the immunosuppressive microenvironment, countering immunosuppression, blocking immune checkpoints, and integrating these approaches with conventional treatments [76]. Studies have shown that pancreatic cancer possesses a uniquely immunosuppressive microenvironment, characterized by limited T lymphocyte infiltration and a relatively low mutational burden. These features contribute to successful immune evasion and facilitate escape from host immune surveillance [77]. To address these challenges, researchers have developed a nanoparticle-based co-delivery system combining MEK/CDK4/6 inhibitors with STING/TLR4 agonists, achieving T cell-mediated control of pancreatic tumors in mice. This approach co-encapsulates innate immune agonists—STING (stimulator of interferon genes) and TLR4 (Toll-like receptor 4)—within lipid nanoparticles alongside RAS-targeted senolytic therapy. It thereby orchestrates type I interferon-driven innate and adaptive immune responses, resulting in durable antitumor efficacy against PDAC [78]. Currently, numerous clinical trials are underway to evaluate the effectiveness of biomarker-driven therapies in pancreatic cancer. For instance, the National Cancer Institute-Molecular Analysis for Therapy Choice (Molecular Analysis for Therapy Choice) trial is a nationwide precision oncology study that assigns patients to targeted treatments based on specific molecular alterations identified in their tumors, independent of tumor histology [79].

Single-cell sequencing is a transformative technology that enables the detection and analysis of genomic, transcriptomic, and epigenomic profiles at the individual cell level. It uniquely captures cellular heterogeneity often obscured in bulk sequencing assays. The application of single-cell sequencing has ushered in a new era for tumor microenvironment (TME) analysis, revealing a pan-cancer blueprint of immune microenvironments, delineating the heterogeneity and differentiation pathways of immune cells, and providing insights for predicting tumor prognosis [80].

In one line of investigation, scRNA-seq of neutrophils was employed to study genetic alterations and underlying processes associated with the Neuregulin expression pattern. Differentially expressed genes were identified, predominantly linked to epidermal development, the apical region of cells, endopeptidase activity, and structural constituents of the extracellular matrix, as determined by Gene Ontology enrichment analysis. Furthermore, Kyoto Encyclopedia of Genes and Genomes pathway analysis indicated that the majority of these genes are involved in pancreatic secretion, protein digestion, and absorption [81].

Other researchers have applied single-cell sequencing to dissect cellular heterogeneity and dynamics across different stages of tumor development. This approach facilitates the identification of mechanisms driving immunosuppression and therapy resistance, thereby aiding in the discovery of potential therapeutic targets [82].

Beyond this, scRNA-seq conducted to analyze tumor evolution and microenvironmental changes before and after chemotherapy has revealed a specific drug-resistant subclone. Based on the characteristics of this subclone, a Genomic Signature for Gemcitabine Sensitivity and Prognosis (GSGP) was constructed, which robustly predicts both sensitivity to gemcitabine and patient prognosis in pancreatic cancer, offering a rationale for clinical personalized treatment strategies [83].

### 4.2. Single-Cell Sequencing in Pancreatic Cancer Precision Diagnostics: Unraveling Potential and Navigating Challenges

Single-cell sequencing is emerging as an instrumental tool for cell biological analysis, with considerable potential to enhance the diagnosis of pancreatic cancer. By enabling the reconstruction of cellular evolutionary trajectories and revealing intratumoral heterogeneity, the integration of single-cell sequencing into the clinical diagnostic workflow for pancreatic cancer appears both feasible and valuable. This approach can overcome the technical limitations of conventional diagnostic methods, thereby providing a more precise and comprehensive basis for clinical decision-making in pancreatic cancer through cutting-edge technology.

Given the subtle early symptoms and rapid progression of pancreatic cancer, conventional diagnostic methods often struggle to detect the disease in its initial stages. Yet in the field of liquid biopsy, single-cell sequencing addresses this challenge effectively: by analyzing circulating tumor cells, it can considerably improve the early detection rate of pancreatic cancer, which in turn facilitates prompt and effective therapeutic interventions for patients [84].

Pancreatic cancer, meanwhile, exhibits pronounced intratumoral heterogeneity, with multiple clonal populations of diverse malignant potential coexisting within the same tumor. Traditional sequencing methods—those that analyze bulk tissue—often fail to resolve these differences; instead, they tend to confuse distinct subtypes, ultimately leading to inaccurate diagnostic classification [85]. In contrast, single-cell sequencing holds a significant advantage: it is capable of providing an in-depth analysis of heterogeneity within pancreatic cancer tumors. The integration of this technology is expected to avoid the misclassification associated with conventional diagnostic methods and offer a more precise and reliable foundation for clinical subtyping. It can be said that with the involvement of single-cell sequencing, clinicians gain more critical insights for assessing tumor subtype and disease severity [86].

Conventional diagnostic methods inadequately characterize the TME of pancreatic cancer. The integration of single-cell sequencing into clinical practice effectively compensates for this gap by enabling a comprehensive assessment of the TME. Studies indicate that single-cell sequencing can identify various subtypes of CAFs. Among these, an inflammatory-like subtype shows increased abundance in pancreatic cancer, which correlates closely with disease progression. This subtype can be quantified via single-cell sequencing and incorporated into diagnostic evaluations [82]. Thus, compared to traditional methods, single-cell sequencing provides critical information for diagnostic staging by resolving the diverse functional cell types within the pancreatic TME, thereby substantially enriching diagnostic data.

Single-cell sequencing holds the potential to uncover novel diagnostic biomarkers for pancreatic cancer, thereby effectively compensating for the limited sensitivity and specificity of conventional markers. Studies indicate that CA19-9, a traditional diagnostic marker, exhibits low specificity for early-stage pancreatic cancer and is susceptible to interference from various factors [87]. In contrast, single-cell sequencing enables the screening for highly specific biomarkers at the individual cell level, offering solutions to the limitations of traditional clinical diagnostics. For instance, analysis of early-stage pancreatic cancer samples via single-cell sequencing has identified specific overexpression of genes such as CLIC4 and GAS2L1 in circulating tumor cells. Notably, the combined detection of GAS2L1 and EPCAM significantly enhances the diagnostic specificity for early-stage disease, markedly outperforming CA19-9 [84]. Therefore, integrating single-cell sequencing into the clinical diagnostic workflow for pancreatic cancer promises to advance and innovate traditional methods, ultimately improving early detection, therapeutic outcomes, and risk management.

## Figures and Tables

**Table 1:** Comparative Analysis of Single-Cell Multi-Omics Technologies in Pancreatic Cancer Research.

Technology Name	Principle	Main Analysis Objects	Technical Advantages (Pancreatic Cancer Applicability)	Disadvantages	Refs.
Single-cell Transcriptome–Epigenome Conjoint Technology (scRNA-seq + scATAC-seq)	Simultaneously detects gene expression and chromatin accessibility in the same single cell; correlates transcription with epigenetic regulation via bioinformatics.	Pancreatic cancer subtype differentiation, CAF plasticity, cancer stem cell epigenetic features	1. Confirms core role of epigenetic regulation in subtype differentiation (data integration efficiency: 93%); 2. Captures epigenetic-gene expression time lag (e.g., 48 h advance in TGF-β/Smad3 pathway).	1. High demand for fresh samples; 2. High computing cost and technical threshold.	[39,40,41,42]
Single-cell Transcriptome–Proteome Concurrent Analysis (CITE-seq)	Antibody-labeled cell surface proteins; synchronously detects gene expression and protein levels.	Immune cell typing, immune checkpoint verification, immunotherapy patient stratification	1. Identifies transcript–protein mismatch (e.g., 67% PD-1 concordance in CD8^+^ T cells); 2. Custom panels detect exhausted T subsets (HR = 2.8, *p* < 0.001).	1. Limited detectable proteins (≤100); 2. Dependent on known biomarkers; 3. Matrix interference risk.	[43,44,45,46]
Spatial Single-Cell Sequencing Technology (10x Visium HD, Nanostring CosMx SMI)	1. Visium HD (10 μm): Spatial gene expression localization; 2. CosMx SMI (1 μm): In situ single-cell gene detection.	Neuroinvasion cell distribution, CAF-immune cell interaction	1. Retains stromal spatial structure; 2. Visium HD: 96% pathological matching; 3. CosMx SMI: Captures 2 μm-range cell signals.	1. Visium HD: Cannot distinguish adjacent cells; 2. CosMx SMI: Limited genes (≤1000); 3. Poor paraffin sample adaptability.	[47,48,49,50]

**Table 2:** Schematic Diagram of Core Functions and Application Outcomes of the Customized Data Analysis Tool for Single-Cell Sequencing of Pancreatic Cancer Cells.

Tool/Algorithm	Core Function	Key Findings	Corresponding to 3.2 Core Technologies	Refs.
SC-RareFind (Rare Cell ID)	Identifies rare cells (<5%) via optimized clustering;	2024 (Genome Biol.): 3.2% CD44^+^CD24^+^ cells (870 k total) identified, 98% accuracy (vs. Seurat: 45% false negative);	Universal (Compatible with all 3.2 technologies)	[56,57]
	2025 upgrade adds CNV for tumor/normal stem cell differentiation	captured ALDH1A1/SOX22025 (Brief. Bioinform.): 100% specificity (20 PDAC samples)		[58,59]
PanCIA (Cell Interaction)	Predicts cell interactions via ligand–receptor (e.g., CellPhoneDB 4.0) + spatial data;	2023 (Nat. Methods): apCAFs recruit T cells (CCL22-CCR4), 5.6x more frequent in immunosuppressive regions (60% higher accuracy vs. traditional tools)	Universal (Focused on spatial single-cell sequencing)	[58,61]
	2024 upgrade adds signaling analysis	2024 (Bioinformatics): M2 macrophages suppress CD8^+^ T (TGF-β/Smad), 91% concordance with co-culture		[59,63]
PanMultiOmics (Multi-Omics Integration)	Integrates transcriptomic/epigenomic/proteomic data via correlation models	2025 (Genome Med.): STAT3 chromatin accessibility vs. phosphorylation (R^2^ = 0.85);	Single-Cell Transcriptome–Epigenome Conjugate Technology, CITE-seq	[60,61]
		co-regulates CAFs → iCAFs (supports STAT3-targeted therapy)		[68]
SingleR	Reference-Based Supervised Cell Type Annotation	Pancreatic cancer cell subpopulation annotation accuracy: 92%, compatible with epithelial/CAFs/immune cell classification	Corresponding to 3.2 Core Technologies	[62,63]
Seurat FindMarkers	Screening of Differentially Expressed Genes (DEGs) at the Single-Cell Level	Efficient identification of TAN-1 subgroup glycolytic-related differentially expressed genes (DEGs) for tumor-mesenchymal cell differential analysis	Corresponding to 3.2 Core Technologies	[62,63]
ClusterProfiler	GO/KEGG/Reactome Pathway Enrichment Analysis	Precise enrichment of pancreatic cancer immune suppression pathways and metabolic pathways, supporting multi-omics DEG joint analysis	Corresponding to 3.2 Core Technologies	[64,65]
ArchR	scRNA-seq + scATAC-seq Data Integration and Trajectory Analysis	Revealing the epigenetic dynamics underlying pancreatic cancer subtype differentiation, with 93% data integration efficiency	Single-Cell Transcriptome–Epigenome Convergence Technology	[65,66]
CiteFuse	CITE-seq Transcript–Protein Data Fusion and Noise Correction	Enhance the accuracy of protein quantification for PD-1/CTLA-4 and other proteins, identifying transcription-protein mismatch subpopulations	CITE-seq	[17,65]
SpatialDE	Spatial Heterogeneity Gene Identification and Localization	Precise capture of the spatial expression patterns of neuroinvasive genes such as TGFBI	Spatial Single-Cell Sequencing (10x Visium HD)	[17,67]
Giotto	Visualization and Analysis of Spatial Cell Interactions	Visualize CAFs-Local Immune Cell Enrichment Patterns, Compatible with 1 μm–10 μm Resolution Data	Spatial Single-Cell Sequencing (General)	[17,69]

## Data Availability

Data sharing is not applicable to this article as no datasets were generated or analyzed during the current study.
